# Effects of an e-Media-Supported, Exercise-Based Phase II Cardiac Rehabilitation in Coronary Artery Bypass Grafting Surgery Patients: A Randomized Controlled Trial

**DOI:** 10.7759/cureus.67557

**Published:** 2024-08-23

**Authors:** Manoj Kumar R, Senthil Kumar T, Balakrishnan Vinod Kumar, Sridevi S, Venkatesh Natarajan

**Affiliations:** 1 Cardiopulmonary Physiotherapy, Sri Ramachandra Institute of Higher Education and Research, Chennai, IND; 2 Cardiology, Sri Ramachandra Institute of Higher Education and Research, Chennai, IND

**Keywords:** quality of life, coronary artery bypass grafting(cabg), cad: coronary artery disease, exercise adherence, functional capacity, e-media supported exercise, telerehabilitation

## Abstract

Background and objective

Coronary artery bypass grafting (CABG) surgery patients undergo cardiac rehabilitation (CR) programs postoperatively to improve their course of recovery. The effectiveness of traditional CR programs is hampered by time constraints, financial burdens, transportation issues, and geographic restrictions. The coronavirus 2019 (COVID-19) pandemic and technological advances have led to the emergence of home-based CR programs using e-media, thereby improving accessibility. This study aimed to analyze the effects of e-media-supported, exercise-based phase II CR in post-CABG patients.

Methods

A single assessor-blinded randomized controlled trial (RCT) was conducted at a tertiary care hospital to analyze the effectiveness of a validated e-media-supported, exercise-based phase II cardiac rehabilitation in CABG Patients. A total of 40 subjects were included in the study based on the inclusion and exclusion criteria. The subjects were then randomly assigned to two groups: the experimental group received e-media-supported exercise and the control group received routine care. The duration of the intervention was three months. The outcome measures used were functional capacity, left ventricular ejection fraction (LVEF), quality of life, and physical activity (PA). Statistical analysis was conducted using SPSS Statistics v. 22.0 (IBM Corp., Armonk, NY).

Results

After three months of intervention, the mean distance covered during the six-minute walk test (6MWT) showed a significant increase in both the control and experimental groups. The experimental group demonstrated a statistically significant improvement compared to the control group (p<0.001). Furthermore, the experimental group showed significant improvements in the rate of perceived exertion (RPE), LVEF, and World Health Organization Quality of Life Brief Version (WHOQOL-BREF) and Global Physical Activity Questionnaire (GPAQ) scores compared to the control group (all p<0.001).

Conclusions

Based on our findings, the e-media-supported, exercise-based phase II cardiac rehabilitation is feasible and safe, and significantly improved functional capacity and enhanced quality of life. The PA level of the experimental group was higher than controls at the 12-week follow-up after CABG.

## Introduction

Coronary artery bypass grafting (CABG) surgery is a widely acknowledged treatment for severe coronary artery disease. However, it is associated with significant postoperative complications such as physical deconditioning, psychological discomfort, and an increased risk of recurrent cardiovascular events [1]. The morbidity and mortality rates within 30 days may be as high as 14.0% and 2.0%, respectively, post-surgery [2]. It has been demonstrated that cardiac rehabilitation (CR) programs, on an individual basis, decrease these risks by promoting cardiovascular fitness, enhancing psychological well-being, and encouraging long-term adherence to heart-healthy behaviors [3,4].

The second phase of CR constitutes the patient's recovery period after a cardiovascular incident or heart surgery. It involves individually tailored, supervised exercise training to build up cardiovascular system strength and general physical endurance. Also, phase II CR provides a wide range of psychological consultations and information about cardiac diseases, the significance of proper nutrition, the use of medications, the recognition of symptoms in cases of complications, and smoking cessation [5]. However, traditional CR programs have several limitations that may discourage patients from participating and adhering to their treatment regimens, especially in rural areas or for those with busy schedules [6]. Cardiac telerehabilitation offers solutions to enhance the uptake of cardiac rehabilitation in women. More flexible, home-based, and smartphone-based models of CR are required to reduce barriers and increase accessibility for women to improve their cardiovascular health [7].

Telerehabilitation is a system of telemedicine for diagnosis, treatment, and remote monitoring, enhancing collaboration among healthcare workers and patients. The coronavirus 2019 (COVID-19) pandemic and the rising influence of the virtual fitness era have led to the emergence of e-media-supported treatment plans as viable alternatives to conventional CR. Virtual systems linked with exercise apps can provide patients with the opportunity for rehabilitation from home using wearable sensors and mobile apps [8]. The e-media-supported programs can enable more flexibility, real-time feedback, and individual adjustments in the exercise regime, thus improving patient adherence and overall results [9]. Studies have shown that home-based CR is as effective as center-based CR in improving patients' quality of life and exercise capacity [10]. Eligible patients undergoing cardiac telerehabilitation experience minimal adverse events during exercise training after a thorough prior evaluation.

We aim to evaluate the effectiveness of e-media-supported interventions in improving the functional capacity and quality of life of CABG patients compared to traditional CR techniques. This study will investigate the potential benefits of e-media-supported CR in enhancing the recovery and long-term prognosis of CABG patients. We believe our findings have the potential to shape future guidelines for postoperative cardiac care, thereby improving the health outcomes of CABG patients worldwide. The study will focus on exercise adherence, as well as the feasibility, safety, and efficacy of e-media-supported phase II CR in CABG patients.

## Materials and methods

Selection of subjects

A single-assessor blinded randomized controlled trial was carried out at a tertiary care hospital from November 2023 to May 2024 to evaluate the effectiveness of e-media-supported, exercise-based phase II cardiac rehabilitation in CABG patients. This study was registered under the clinical trial registry with the trial number CTRI/2024/01/061301. Based on the sample size calculation using the software nMaster version 2.0, the study included 40 subjects. The sampling was randomized using a restricted block randomization method, with each block having a size of 10.

The study included adult patients aged between 35 and 65 who had undergone traditional CABG surgery, patients referred by the cardiologist for phase II cardiac rehabilitation, patients with low cardiac risk [based on American Association of Cardiovascular and Pulmonary Rehabilitation (AACVPR) guidelines], and patients with access to electronic media devices and internet connectivity. Patients with significant cognitive impairments or mental health issues, patients with major comorbidities or health problems, patients with contraindications to exercise or significant medical conditions, and individuals who had participated in an organized CR program in the previous six months were excluded from the study.

Procedure

The research involved the following 5 steps: (1) Develop an exercise rehabilitation training process supported by electronic media. (2) Validate the content of the developed training program. (3) Incorporate the contents into e-media tools for rehabilitation programs. (4) A pilot study to establish the clinical safety and feasibility (5). The randomized controlled trial (RCT) to evaluate the effectiveness of phase II cardiac rehabilitation was developed based on electronic means.

Step 1: Develop an Exercise Rehabilitation Training Process Supported by Electronic Media

The e-media-supported exercise program is a structured rehabilitation program that includes components and parameters for training and progression. It was developed based on an analysis of the existing literature on cardiac rehabilitation of patients after cardiac surgery, American Heart Association (AHA)/American Association of Cardiovascular and Pulmonary Rehabilitation (AACVPR), American College of Sports Medicine (ACSM) guidelines, and expert input.

The duration of exercise training is 12 weeks. It consists of aerobic exercise for the first four weeks, followed by a month of combined resistance and aerobic training. The final four weeks prioritize flexibility training along with strength and aerobic exercises. The exercise prescriptions follow the Frequency, Intensity, Time, and Type (FITT) principle of ACSM (Table 1) [11].

**Table 1:** FITT principle of prescribed exercises as per ACSM guidelines ACSM: American College of Sports Medicine; FITT: Frequency, Intensity, Time, and Type; reps: repetition; RM: repetition maximum; RPE: rate of perceived exertion

Exercise Protocol	First two weeks	Second two weeks	Third two weeks	Fourth two weeks	Fifth two weeks	Sixthtwo weeks	
								Aerobics						
Frequency	5 days/week	5 days/week	5 days/week	5 days/week	5 days/week	5 days/week	
Intensity	RPE 11-13 on a 6 to 20 scale	RPE 11-13 on a 6 to 20 scale	RPE 11-13 on a 6 to 20 scale	RPE 11-13 on a 6 to 20 scale	RPE 11-13 on a 6 to 20 scale	RPE 11-13 on a 6 to 20 scale	
Duration	25 minutes	30 minutes	40 minutes	40 minutes	50 minutes	50 minutes	
			Resistance				
Frequency			2-3 nonconsecutive days/week	2-3 nonconsecutive days/week	2-3 nonconsecutive days/week	2-3 nonconsecutive days/week	
Intensity			40% 1 RM	50% of 1 RM	50% of 1 RM	60% of 1 RM	
Duration			10 reps/set, 2 sets	10 reps/set, 2 sets	10 reps/set, 3 sets	10 reps/set, 3 sets	
					Flexibility		
Frequency					5 days/week	5 days/week	
Intensity					Tolerance level	Tolerance level	
Duration					15-second hold/set, 3 sets	15-second hold/set, 4 sets	

Step 2: Validate the Content of the Developed Training Program

This step involves validating the exercise program developed for e-media-supported, exercise-based phase II cardiac rehabilitation in CABG patients using the content validation format [12]. The content validation form was created to ensure that experts in cardiovascular rehabilitation carefully evaluated and provided feedback on the developed exercises. The validation was obtained from one interventional cardiologist and four physiotherapists specialized in cardiorespiratory. Their ratings resulted in a Content Validity Index (CVI) score of 0.94, indicating that the material has been validated for the use of patients.

Step 3: Incorporate the Contents Into E-Media Tools for Rehabilitation Programs

Incorporate exercise videos and educational materials into e-media tools for rehabilitation programs, including detailed exercise descriptions, safety precautions, self-monitoring guidelines, and bilingual accessibility in Tamil and English.

Step 4: A Pilot Study to Establish the Clinical Safety and Feasibility

The study included 10 participants who were chosen using the inclusion and exclusion criteria. They received consent forms and information about the study. They were provided with study details and guidelines, including the use of a WhatsApp channel for communication. The trial lasted six weeks, and pre- and post-training data were collected using the six-minute walk test. The post-training results showed a significant improvement, demonstrating the effectiveness of the developed protocol.

Step 5: RCT to Evaluate the Effectiveness of Phase II Cardiac Rehabilitation Developed Based on Electronic Means

**Figure 1:**
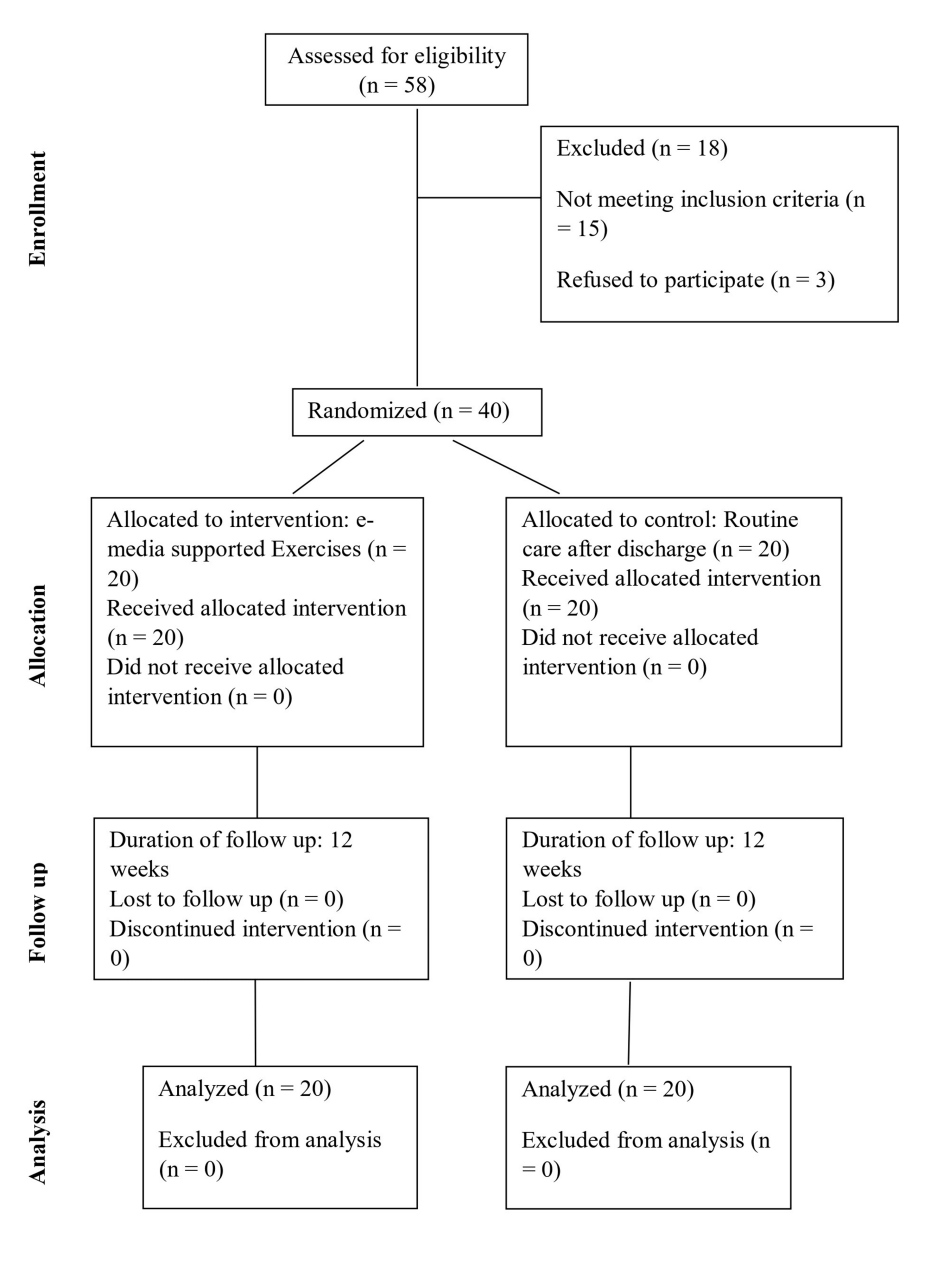
CONSORT diagram showing the flow of participants through each stage of a randomized trial CONSORT: Consolidated Standards of Reporting Trials

A single assessor-blinded RCT was conducted based on the Consolidated Standards of Reporting Trials (CONSORT) protocol (Figure 1). Participants who provided consent were randomly divided into two groups: the experimental group and the control group. The control group received standard care, while the experimental group participated in e-media-supported exercises via Google Meet and WhatsApp. Baseline data was collected, and the intervention lasted for three months.

Intervention

e-Media-supported Exercise: Experimental Group

The experimental group was trained with the developed protocol using e-media. Before discharge, patients were educated on access to a WhatsApp channel-tailored platform for the study of delivering e-media-supported exercise in phase II cardiac rehabilitation. Self-demonstrated videos shared through a WhatsApp channel delivered the exercise protocol, along with educational materials, safety precautions, and training benefits. Additionally, the face-to-face virtual approach via Google Meet was used to explain the training exercise protocols, their benefits, uses, and safety precautions that need to be considered during exercise. Follow-ups were conducted weekly to monitor progress. Participants were taught to use a pulse oximeter and RPE scoring and encouraged to regularly update their vital signs and RPE via WhatsApp. Each participant received a tailored exercise plan based on their RPE score.

Routine Care: Control Group

The control group received standard postoperative care, which included patient counseling on lifestyle modifications such as smoking cessation and reducing alcohol intake. Patients were educated on the importance of maintaining regular physical activity (PA) tailored to their recovery stage to support cardiovascular health. In addition to lifestyle counseling, the standard care included dietary recommendations focused on heart-healthy eating patterns. As part of the home care instructions, patients were educated on how to monitor their vital signs, such as heart rate and blood pressure, and recognize symptoms that might indicate complications, including shortness of breath, chest pain, or swelling in the legs. Patients were also instructed on wound care, including keeping the surgical site clean and dry, recognizing signs of infection, and avoiding strenuous activities that could strain the incision. They were advised to adhere to prescribed medications, attend follow-up appointments, and report any unusual symptoms to their healthcare provider immediately.

Outcome measures

The outcome measures used in the study were the six-minute walk test (6MWT), rate of perceived exertion (RPE), for functional capacity, left ventricular ejection fraction (LVEF), World Health Organization Quality of Life Brief Version (WHOQOL-BREF) for quality of life, and Global Physical Activity Questionnaire (GPAQ) for PA.

The Six-Minute Walk Test (6MWT)

The six-minute walk test is a measure that calculates the so-called functional exercise capacity of a patient by measuring the distance he or she can walk in six minutes, following the American Thoracic Society (ATS) criteria. It is a common measure in cardiac rehabilitation.

The Borg RPE Scale

The Borg RPE Scale is a subjective tool to determine an individual’s level of exertion. Typically, it ranges from 6 to 20, where 6 represents no effort and 20 represents maximum effort. During the six-minute walking test, participants rate their perceived exertion at different points during the test to provide insights into their level of exertion and fatigue.

Left Ventricular Ejection Fraction (LVEF)

LVEF is an important indicator of cardiovascular health. It is typically measured by cardiologists using M-mode and two-dimensional (2D) echocardiography. It shows the amount of blood pumped by the left ventricle with each heartbeat. This is an important indicator of cardiovascular health, with normal values usually ranging from 50% to 70%.

World Health Organization Quality of Life Brief Version (WHOQOL-BREF)

The WHOQOL team worked with 15 global centers to develop the WHOQOL to provide a quality-of-life assessment that can be used in a variety of cross-cultural contexts. The shortened version of the WHOQOL-BREF questionnaire has two items assessing general health and overall quality of life and 24 items measuring satisfaction in four different domains: physical health (DOM1), which has seven items; psychological health (DOM2), which has six factors; social relationships (DOM3), which has three factors; and environmental health (DOM4), which has eight factors.

Global Physical Activity Questionnaire (GPAQ)

The WHO created the GPAQ in 2002 as part of the STEPwise approach to monitoring chronic disease risk factors to observe PA. In 2004, as part of the Global Strategy on Diet, Physical Activity, and Health, the WHO recommended its use for national PA surveillance. It consists of 16 items designed to assess a person's level of PA in three domains: work, transportation, and recreation, in addition to time spent engaging in sedentary behavior (SB).

## Results

The statistical analysis was carried out using SPSS Statistics v. 22.0 (IBM Corp., Armonk, NY). The study's confidence interval was set at 95%, with a significance level of p=0.05. The Shapiro-Wilk test was used to determine the normality of the data. Descriptive statistics were used to describe the baseline demographics (Table 2). The t-test was used to evaluate hypotheses about normally distributed data (distance traveled in 6MWT). The paired t-test was used to compare pre-and post-test values in the experimental and control groups, whereas the independent t-test was used to compare pre- and post-test values across groups. Non-parametric tests were employed to analyze non-normally distributed data such as RPE, WHOQOL-BREF, LVEF, and GPAQ scores. The Wilcoxon signed-rank test was used to examine pre- and post-test values within groups, while the Mann-Whitney U test was employed to analyze between-group differences.

**Table 2:** Baseline demographics of both groups ^*^Mann-Whitney U test BMI: body mass index; SD: standard deviation

Variables	Control group	Experimental group	P-value^*^
Age, years, mean ±SD	55.94 ±6.91	57.75 ±4.84	0.23
Height, cm, mean ±SD	163.2 ±7.22	162.6 ±6.09	0.81
Weight, Kg, mean ±SD	69.2 ±8.81	68.65 ±9.19	0.85
BMI, Kg/m^2^,mean ±SD	26.31 ±2.61	25.8 ±3.04	0.70
Systolic BP, mmHg, mean ±SD	127.6 ±11.95	126.2 ±11.01	0.64
Diastolic BP, mmHg, mean ±SD	78.55 ±7.63	79.05 ±8.55	0.99
Ejection fraction, %, mean ±SD	58.1 ±5.345	57.15 ±5.15	0.84
Male, n (%)	18 (80%)	18 (80%)	0.50
Female, n (%)	2 (20%)	2 (20%)	0.50
Diabetes, n (%)	14 (70%)	16 (80%)	0.29
Hypertension, n (%)	14 (70%)	13 (65%)	0.39
Smoker, n (%)	11 (55%)	12 (60%)	0.49
Non-smoker, n (%)	9 (45%)	8 (40%)	
Alcoholic, n (%)	13 (65%)	14 (70%)	0.42
Non-alcoholic, n (%)	7 (35%)	6 (30%)	

Both the control and experimental groups significantly improved their distance covered during 6MWT (p<0.001) (Table 3). The experimental group showed statistically significant improvement over the control group (p<0.001) (Table 4).

**Table 3:** Comparison of pre-training and post-training values of distance covered in 6MWT within the control and experimental groups ^*^Paired t-test 6MWT: six-minute walk test; SD: standard deviation

Groups	Pre-training, meters	Post-training, meters	P-value^*^
	Mean ±SD	Mean ±SD	
Control	265.25 ±11.86	323.25 ±16.56	<0.001
Experimental	264 ±12.2	460 ±20.38	<0.001

**Table 4:** Comparison of pre-training and post-training values of distance covered in 6MWT between the control and experimental groups ^*^Unpaired t-test 6MWT: six-minute walk test; SD: standard deviation

	Control	Experimental	P-value^*^
	Mean ±SD	Mean ±SD	
Pre-training, meters	265.25 +11.86	264 +12.2	0.744
Post-training, meters	323.25 +16.56	460 +20.38	<0.001

The mean RPE values for both groups differed significantly from pre-test to post-test (p<0.001) (Table 5). Between-group analysis revealed that the experimental group had a considerably higher reduction in RPE scores than the control group (Table 6).

**Table 5:** Comparison of pre-training and post-training RPE scores in 6MWT within the control and experimental groups ^*^Wilcoxon signed-rank test 6MWT: six-minute walk test; RPE: rate of perceived exertion; SD: standard deviation

Groups	Pre-training	Post-training	P-value^*^
	Mean ±SD	Mean ±SD	
Experimental	13.7 ±1.13	7.65 ±0.81	<0.0001
Control	13.15 ±0.87	10.4 ±0.99	<0.0001

**Table 6:** Comparison of pre-training and post-training RPE scores in 6MWT between the control and experimental groups ^*^Mann-Whitney U test 6MWT: six-minute walk test; RPE: rate of perceived exertion; SD: standard deviation

	Experimental	Control	P-value^*^
	Mean ±SD	Mean ±SD	
Pre-training	13.7 ±1.13	13.15 ±0.87	0.097
Post-training	7.65 ±0.81	10.4 ±0.99	<0.0001

The WHOQOL-BREF, which measures the quality of life across four dimensions, showed significant improvement from the pre-test to the post-test (p<0.001) (Table 7). Between-group analysis revealed that the experimental group improved their quality-of-life scores significantly more than the control group in the post-test values (Table 8).

**Table 7:** Comparison of pre-training and post-training WHOQOL-BREF scores within the control and experimental groups ^*^Wilcoxon signed-rank test SD: standard deviation; WHOQOL-BREF: World Health Organization Quality of Life Brief Version

Variables		Pre-training	Post-training	P-value^*^
	Groups	Mean ±SD	Mean ±SD	
QOL Physical domain	Experimental	29.34 ±6.55	28.57 ±5.95	<0.0001
	Control	29.05 ±5.95	58.03 ±6.33	
QOL Psychological domain	Experimental	32.01 ±5.86	88.88 ±5.14	<0.0001
	Control	32.67 ±6.11	59.36 ±8.1	
QOL Social domain	Experimental	35.15 ±3.92	79.38 ±4.26	<0.0001
	Control	35.39 ±4.57	59.57 ±7.29	
QOL Environmental domain	Experimental	30.07 ±6.97	85.58 ±3.38	<0.0001
	Control	29.92 ±7.57	54.06 ±3.38	

**Table 8:** Comparison of pre-training and post-training WHOQOL-BREF scores between the control and experimental groups ^*^Mann-Whitney U test SD: standard deviation; WHOQOL-BREF: World Health Organization Quality of Life Brief Version

Variables		Experimental	Control	P-value^*^
		Mean ±SD	Mean ±SD	
QOL Physical domain	Pre-training	29.34 ±6.55	29.05 ±5.95	0.659
	Post-training	80.35 ±3.37	58.03 ±6.33	<0.0001
QOL Psychological domain	Pre-training	32.01 ±5.86	32.67 ±6.11	0.862
	Post-training	88.88 ±5.14	59.36 ±8.1	<0.0001
QOL Social domain	Pre-training	35.15 ±3.92	35.39 ±4.57	0.398
	Post-training	79.38 ±4.26	59.57 ±7.29	<0.0001
QOL Environmental domain	Pre-training	30.07 ±6.97	29.92 ±7.57	0.583
	Post-training	85.58 ±3.38	54.06 ±3.38	<0.0001

The median LVEF values for both groups improved significantly from pre-training to post-training (p<0.001) (Table 9). Between-group analysis demonstrated that the experimental group improved their LVEF significantly more than the control group in the post-test values (Table 10). In addition, the experimental group had considerably higher post-test GPAQ scores than the control group (Table 11).

**Table 9:** Comparison of pre-training and post-training LVEF values within the control and experimental groups ^*^Wilcoxon signed-rank test LVEF: left ventricular ejection fraction; SD: standard deviation

Groups	Pre-training	Post-training	P-value^*^
	Mean ±SD	Mean ±SD	
Experimental	51.3 ±3.54	59.05 ±2.37	<0.0001
Control	51 ±3.42	55.35 ±2.81	<0.0001

**Table 10:** Comparison of pre-training and post-training LVEF values between the control and experimental groups ^*^Mann-Whitney U test LVEF: left ventricular ejection fraction; SD: standard deviation

	Experimental	Control	P-value^*^
	Mean ±SD	Mean ±SD	
Pre-training	51.3 ±3.54	51 ±3.42	0.097
Post-training	59.05 ±2.37	55.35 ±2.81	<0.0001

**Table 11:** Comparison of post-training GPAQ scores between the control and experimental groups ^*^Mann-Whitney U test GPAQ: Global Physical Activity Questionnaire; SD: standard deviation

Group	Post-training	P-value^*^
	Mean ±SD	
Control	546 ±153.15	<0.0001
Experimental	1350 ±446.34	

## Discussion

Results of this randomized controlled trial suggest that an e-media phase II exercise-based cardiac rehabilitation program offers better outcomes for postoperative CABG patients than traditional rehabilitation methods. In the trial group, multiple domains of outcome manifested considerable improvements in functional capacity, exercise rate, quality of life, and LVEF. Moreover, it has been shown to have high exercise adherence and completion levels in the experimental group, with an average of 93.65 ±4.51% for exercise adherence. Heart rate, oxygen saturation, exercise rate, and recovery time in each stage of the rehabilitation program showed improvements, as the descriptive statistics have consistently proven. At any instance during the trial or after it, there was no evidence of adverse events or safety concerns, which showed the safety and efficacy of e-media-supported exercises.

These findings are in line with studies that support the value and potential of electronic-supported rehabilitation programs. Taylor et al. (2022) stressed the value of having alternate models of the said CDs at a time when accessibility to their traditional programs is limited worldwide and, in particular, in low-income regions of the world. This review, therefore, underpins the role of technology-based and home-based programs in filling this gap, which our study finds could be the case with electronically supported CR in terms of enhancing engagement, participation, and outcomes [13].

Dalal et al. (2021) conducted a Cochrane review that covered the evidence for the growth in digitally delivered, home-based models of CR programs. It highlighted that the COVID-19 pandemic has accelerated the adoption rates of these models and has offered an unprecedented opportunity to reimagine how CR is delivered. The study concludes that e-media can achieve effective motivation, guidance, and adherence to exercise programs [14]. Taylor et al. (2022) focused on the promise of tele-cardiac rehabilitation in increasing participation in CR, particularly during the pandemic. The results showed that home-based cardiac telerehabilitation (HBCTR) was as effective as having no CR and similar to in-center options; however, they identified several challenges to be addressed for telerehabilitation to be effectively implemented. This study adds to this evidence by showing that electronically supported CR can indeed improve outcomes, although further research is needed to optimize its integration into the services now available [15].

Mujeeb and Kazi (2020) concluded that home-based and center-based cardiac rehabilitation were equally effective in improving functional capacity and left ventricular function in CABG patients [16]. Similarly, an RCT by Dorje et al. (2019) evaluated the effectiveness of a cardiac telerehabilitation program mediated by a smartphone application using the WeChat platform for delivering and monitoring exercise training in patients after percutaneous coronary intervention. It was found to be very effective, accessible, and easy to use, significantly improving functional capacity and secondary prevention of coronary artery disease [17]. Foccardi et al. (2021) did a pilot RCT to test the effectiveness of reminders via SMS in promoting physical interest adherence after CR. They determined that such reminders represent a cost-efficient way to enhance adherence, doubtlessly improving cardiovascular health outcomes [18]. Wang et al. (2020) reported that WeChat-based absolute intervention improved treatment adherence and encouraged constant bodily interest in post-CABG patients, leading to enhancements in low-density lipoprotein cholesterol levels and systolic BP [19].

The marked improvement in parameters found in this study, alongside evidence from other studies, suggests that e-media can promote program adherence among patients by making it feasible and safe to use. The exercise routines are more effective, principally due to their superior overall performance effects. E-media systems can provide convenient and engaging platforms for delivering rehabilitation programs, which can enhance patient participation and health-related outcomes.

Limitations of the study

The study's sample group predominantly consisted of males, due to the lower prevalence of CABG among females. A cost-benefit analysis should be conducted to assess the economic viability of implementing the program on a larger scale for electronically assisted rehabilitation.

Future scope of study

Future studies should include a more extensive group of female subjects to determine the feasibility of electronically supported phase II CR. This could involve integrating advanced technologies such as specific software, applications, or sensors for self-monitoring during and after exercise. Additionally, future research should focus on customizing virtual media rehabilitation programs to fit individual needs, preferences, and abilities. Tailored programs have the potential to optimize the effectiveness and intensity of participation. Longitudinal studies are needed to evaluate the long-term effectiveness and sustainability of e-media-based CR.

## Conclusions

Based on our findings, the e-media-supported, exercise-based phase II cardiac rehabilitation is feasible, effective, safe, and easy to implement in practice for CABG patients. This approach significantly improves functional capacity and quality of life. Rehabilitation supported by electronic media provides a means of safe and effective phase II training, overcoming the barriers to participation in rehabilitation and providing an alternative to institution-dependent cardiac care after surgery.
